# The feasibility of robotic navigation in single position oblique lateral spine surgery. A technical note and a retrospective study

**DOI:** 10.3389/fsurg.2025.1582889

**Published:** 2025-06-25

**Authors:** Asrafi Rizki Gatam, Luthfi Gatam, Ajiantoro Ajiantoro, Omar Luthfi, Phedy Phedy, Harmantya Mahadhipta, Syafruddin Husin, Karina Sylvana Gani, Mitchel Mitchel, Erica Kholinne

**Affiliations:** ^1^ Department of Orthopedics, Gatam Institute, Tangerang, Indonesia; ^2^ Department of Orthopedic Surgery, Fatmawati Hospital, Jakarta, Indonesia; ^3^ Department of Orthopedic Surgery, Premier Bintaro Hospital, Tangerang, Indonesia; ^4^ Department of Orthopedic Surgery, Faculty of Medicine, Universitas Trisakti, Jakarta, Indonesia

**Keywords:** robotic-assisted, OLIF, minimal invasive surgery, single position, surgical technique

## Abstract

**Introduction:**

Robotic-assisted techniques in minimally invasive spine surgery are recognized for their potential to enhance surgical precision, minimize intraoperative complications, and improve clinical outcomes. A significant advantage of robotics in oblique lateral interbody fusion (OLIF) is the capability to perform single-position surgery, allowing simultaneous anterior and posterior procedures without the need to reposition the patient.

**Methods:**

A retrospective review of 25 consecutive patients who underwent robotic-navigated single-position OLIF spine surgery was performed. Data collected included back and leg pain scores (VAS), screw placement accuracy, operative time, estimated blood loss, postoperative length of stay, and surgical complications.

**Results:**

In total, 116 screws were placed robotically in 25 patients, with a mean age of 62.2 ± 8.9 years. Diagnoses included grade 1 (10 patients) or grade 2 (7 patients) spondylolisthesis and degenerative disc disease (8 patients). The mean operative time from incision to closure was 101.2 ± 7.2 min, with an estimated intraoperative blood loss of 90.0 ± 16.6 ml. VAS scores for leg and back pain improved from preoperative to six months postoperative (from 3.6 to 1 for leg pain and 5.3 to 1 for back pain). Two major vein complications and one retrograde ejaculation.

**Conclusion:**

Single-position OLIF shows promising results, with robotic guidance offering substantial benefits, including reduced bleeding, fewer surgical complications, and shorter operative times, all without flipping the patient. Robotic assistance in OLIF holds great potential and broad application prospects in spine surgery.

## Introduction

Spine surgery has significantly evolved over the past decade, moving from conventional open procedures to minimally invasive spine surgery, which has now become the standard approach for managing various spinal pathologies (1). Minimally invasive spine surgery reduces soft tissue damage around the surgical site, resulting in smaller incisions, decreased blood loss, and quicker recovery to normal activities (1, 2). Surgeons utilize percutaneous pedicle screws and specialized posterior or lateral lumbar retractors to accomplish these objectives. While minimally invasive surgery has shown favorable outcomes, it should be noted that there is radiation exposure to patients, surgeons, and operating room personnel (3).

Robotic navigation in spine surgery addresses concerns about radiation exposure by enabling real-time instrument and pedicle screw tracking without radiation (4). Spinal surgery is complex because it is close to nerves and major blood vessels, making precision and accuracy essential. Robotic assistance can enhance accuracy and minimize human error (5).

Lateral lumbar surgery is one of the surgical techniques that is benefited by robotic navigation technology. Traditionally, surgeons need to flip the patient from the lateral decubitus position to the prone after the implantation of the interbody cage for fusion; this flip will lengthen the surgical time, increase patient exposure to the anesthetic agent, and increase hospital operating room time, which could increase the total unit cost. Recently, the Oblique Lateral Interbody Fusion (OLIF) technique has gained attention for its advantages, particularly its retroperitoneal approach, which eliminates the need to divide the psoas major muscle while reducing the risk of injury to major vessels, the ureter, and the lumbar plexus.

The current retrospective study aimed to investigate the feasibility of a robot-assisted OLIF procedure for degenerative disc disease of the lumbar spine.

## Material and methods

This is a retrospective cohort study of 25 patients who underwent an oblique lateral interbody fusion with robotic navigation percutaneous screw fixation. A team of four spine surgeons performed all surgeries from September to December 2022. All data were obtained through the extraction of inpatient medical records.

The inclusion criteria were: (1) Patients diagnosed varied from grade 1 or 2 degenerative spondylolisthesis and degenerative disc disease. (2) Patients with conservative treatment showed no significant effects after 3–6 months.

We evaluate clinical outcomes after surgery in the form of visual analog scale (VAS) back pain and leg pain, the screw accuracy, operating time, estimated blood loss, postoperative length of stay (LOS), and complications after surgery. The VAS 0 indicated no pain, and 10 represented the worst pain. These scores were collected preoperatively, one, three, and six months post-surgery. Screw accuracy was assessed using a post-operative CT scan. The operative time was counted as the interval from the initial skin incision to the skin closure. Estimated blood loss (EBL) was measured intraoperatively from a suction bottle and gauze. Postoperative LOS was the hospitalization period from hospital administration to postoperative without the need for analgesics. Complications were measured intraoperative, postoperative, and during follow-up time.

### Surgical technique

#### Registration

The ExclesiusGPS robotic platform (Globus Medical; Audubon, PA, USA) is securely attached to both the bed and the patient's spine at the posterior superior iliac spine. Two fluoroscopic images align the patient's anatomical position with the preoperative segmented CT images. It is crucial to minimize any patient movement to avoid errors in the registration process.

### Anesthesia and position

**Figure 1:**
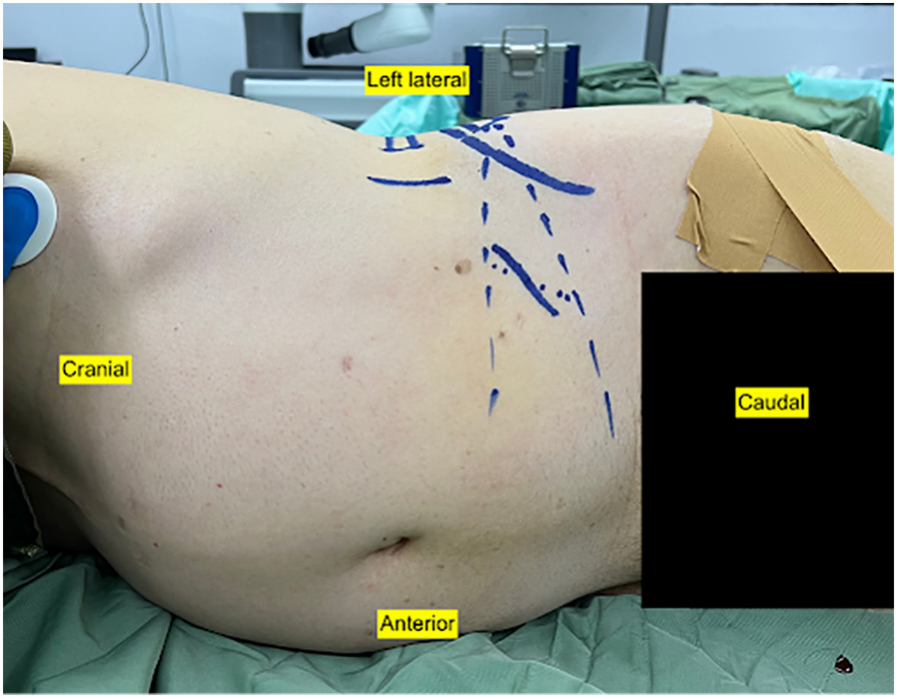
The patient was positioned at the lateral decubitus with the left side up.

The patient is positioned in the lateral decubitus position with the left side up under general anesthesia (Figure 1). This position allowed the surgeons to work simultaneously from anterior and posterior, OLIF from the anterior side, and percutaneous robotic navigated screw from the posterior side.

### Software planning

**Figure 2:**
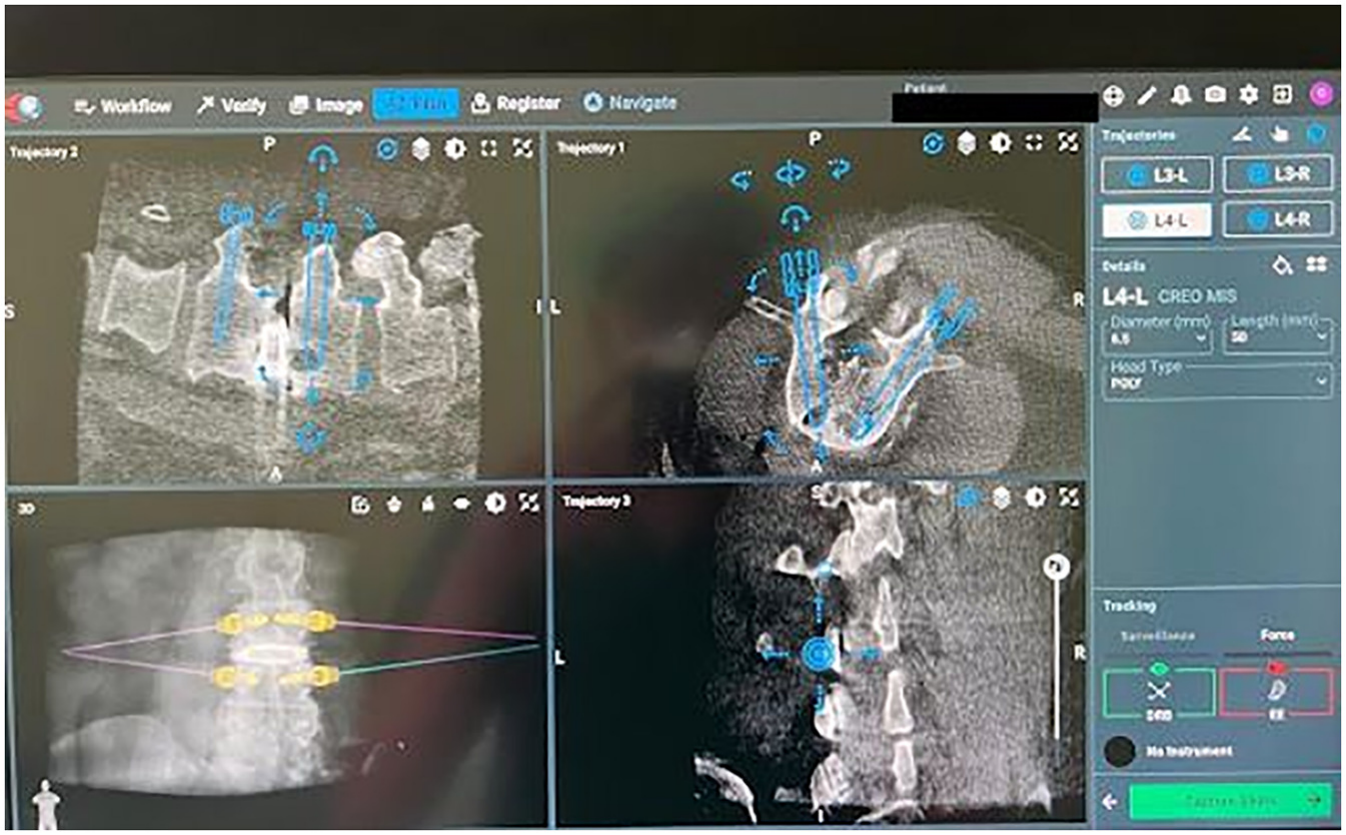
ExcelsiusGPS software screw planning includes entry points, trajectories, screw length, and width.

The ExcelsiusGPS planning software sets targets and designs construct trajectories based on the patient's preoperative or intraoperative CT scans. Successful planning with the CT scans and the ExcelsiusGPS system includes meticulous instrument registration and precise screw trajectory mapping to minimize potential complications (Figure 2).

### Posterior procedure

**Figure 3:**
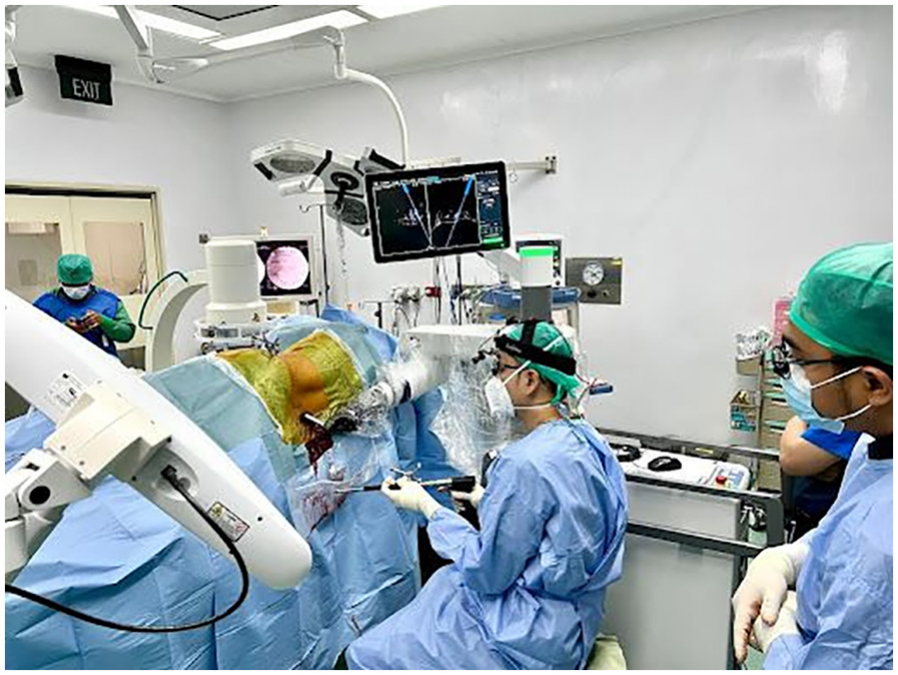
Posterior side showing pedicle screw insertion assisted by a robotic arm.

Insertion of the percutaneous posterior screw was navigated by the robotic system (Figure 3). We implemented a preoperative workflow that involved registering the preoperative CT scan into the Excelsius GPS system and merging the intraoperative C-arm images with the CT scan. Dynamic reference frames and surveillance markers were affixed to the iliac wing to prevent obstruction during procedures, whether utilizing the robot or performing the OLIF. A single midline skin incision was made. The robotic-assisted technique for screw placement initiates with the insertion of a robotic knife down to the bone, followed by the navigated dilator and cannula. Subsequently, the navigated drill, tap, and pedicle screw are advanced along the robotic arm. Screw placement is performed from proximal to distal to enhance accuracy and minimize errors. The right side is addressed first to facilitate the downward drainage of any incisional bleeding; starting with the left side could lead to obscuration of surgical field by blood later on. Once all screws were positioned, the anterior surgeon performed the discectomy as decompression procedure and cage placement. After completing these steps, the pedicle screws were then secured.

### Anterior procedure

**Figure 4:**
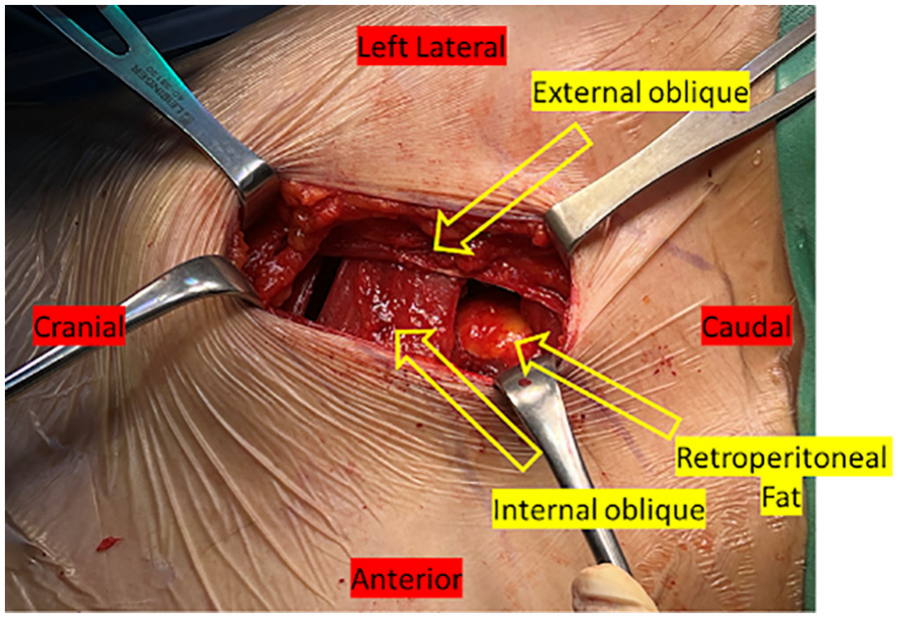
An intraoperative image displays retroperitoneal fat visible following the division of the muscles and fascia.

**Figure 5:**
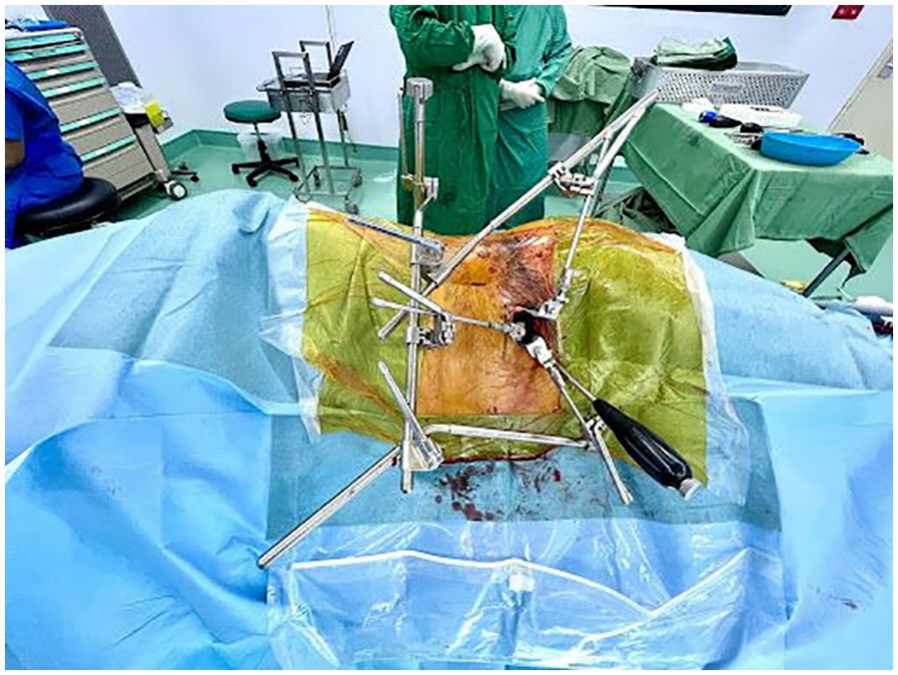
Anterior side showing surgical corridor with three minimally invasive retractor blades.

**Figure 6:**
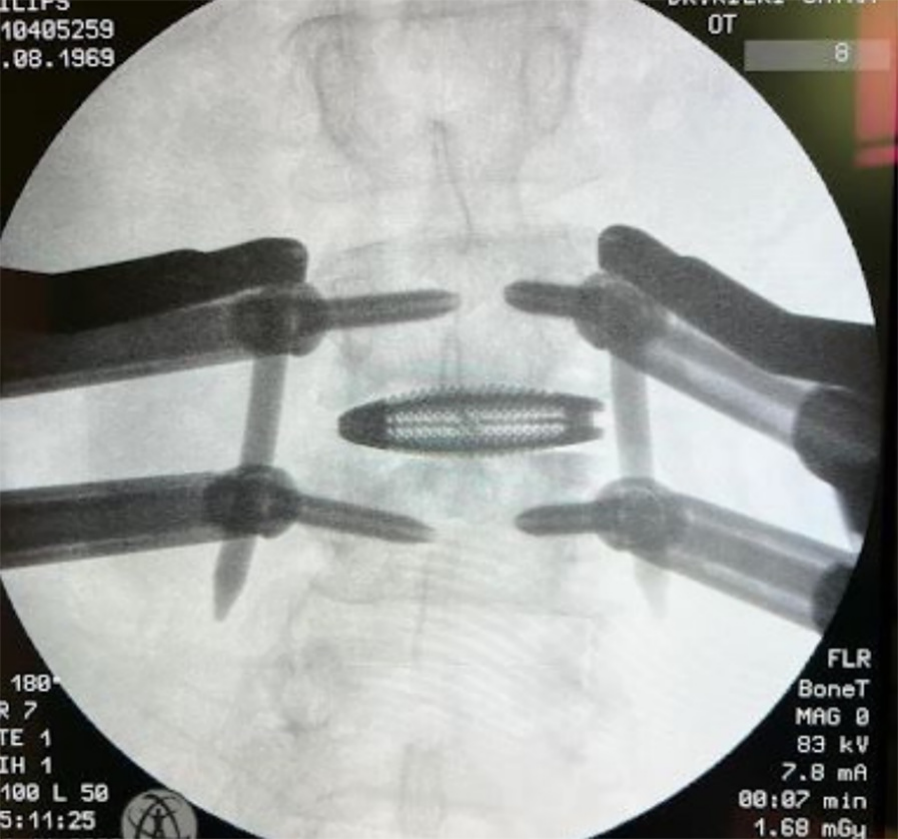
The intraoperative C-arm image displays the interbody cage placement. The implant should span the entire apophyseal ring.

The robotic arm is aligned with pre-planned disc spaces, marking the skin to guide the surgical corridor. In OLIF, incisions were placed 5–7 cm from the center of the disc, depending on the level of the pathology. External oblique, internal oblique and transversal fascia were split until we saw the peritoneum and retroperitoneal fat (Figure 4). The peritoneum was released from surrounding tissue by finger dissection until we could palpate the psoas muscle. The robotic arm assists in posterior screw placement while the anterior surgeon exposes the disc spaces. It is crucial to avoid patient movement to prevent errors in robotic guidance. If the posterior surgeon is still working, the L5-S1 disc is exposed first through an oblique corridor, followed by L4–5 and L3–4. Once screw placement is complete, the anterior surgeon proceeds with discectomy, dilating, and the cage is inserted in a standard OLIF fashion (Figures 5, 6). Closure begins simultaneously as the posterior surgeon finishes.

## Results

A total of 25 patients were included in the study. The diagnosis was grade 1 spondylolisthesis (10 patients), grade 2 spondylolisthesis (7 patients), and degenerative disc disease (8 patients). Their mean age was 62.2 years old (range 43–82 years), with an average follow-up of 29.6 ± 1.6 weeks after surgery (Table 1). The mean preoperative VAS of leg pain was 3.6, and the mean VAS of back pain was 5.3. The outcomes of this study are as follows; further details can be found in Table 2.

**Table 1:** Demographic data.

Case No.	Age (years)	Gender (M/F)	Diagnosis	Segment	Follow-up time (weeks)
1	64	M	Degenerative disc disease	L3-5	30.0
2	67	M	Degenerative disc disease	L3-5	30.0
3	68	M	Degenerative disc disease	L3-5	31.3
4	69	F	Degenerative disc disease	L2-4	27.8
5	72	F	Degenerative disc disease	L1-4	30.0
6	76	F	Degenerative disc disease	L4-S1	26.9
7	79	M	Degenerative disc disease	L3/4	28.2
8	82	F	Degenerative disc disease	L3/4	31.7
9	43	M	Grade 1 spondylolisthesis	L3/4	26.5
10	46	F	Grade 1 spondylolisthesis	L3/4	31.3
11	55	F	Grade 1 spondylolisthesis	L5/S1	30.9
12	56	F	Grade 1 spondylolisthesis	L4/5	27.4
13	59	M	Grade 1 spondylolisthesis	L5/S1	30.0
14	59	F	Grade 1 spondylolisthesis	L5/S1	30.4
15	59	F	Grade 1 spondylolisthesis	L4/5	30.9
16	59	F	Grade 1 spondylolisthesis	L5/S1	30.4
17	61	F	Grade 1 spondylolisthesis	L3/4	30.0
18	63	F	Grade 1 spondylolisthesis	L5/S1	31.7
19	51	M	Grade 2 spondylolisthesis	L4/5	29.5
20	58	F	Grade 2 spondylolisthesis	L3/L4	29.5
21	61	M	Grade 2 spondylolisthesis	L3/4	27.8
22	61	M	Grade 2 spondylolisthesis	L4/5	30.9
23	62	M	Grade 2 spondylolisthesis	L5/S1	29.5
24	62	M	Grade 2 spondylolisthesis	L5/S1	29.5
25	63	F	Grade 2 spondylolisthesis	L2/3	26.9
Mean ± SD	62.2 ± 8.89				29.6 ± 1.6

M, male patient; F, female patient.

**Table 2:** Surgical data and clinical outcomes.

Case No.	EBL (ml)	Operative time (min)	Preoperative VAS		Postoperative VAS leg pain			Postoperative VAS back pain			Hospital stay (days)
			Leg pain	Back pain	1 month	3 months	6 months	1 month	3 months	6 months	
1	80	99.7	3	5	2	1	1	2	2	1	3
2	50	106.8	3	5	2	1	0	2	3	1	4
3	100	91.7	5	6	2	1	1	2	2	1	3
4	100	113.7	3	5	2	1	1	2	2	1	4
5	70	107.9	3	6	2	2	2	2	2	1	3
6	85	100.9	6	7	2	2	1	2	2	0	3
7	90	89.9	2	5	2	0	0	2	2	1	3
8	120	99.8	6	6	2	1	0	2	2	1	4
9	90	88.7	2	5	2	2	1	2	2	0	2
10	95	110.1	4	4	2	2	2	2	2	1	3
11	100	99.3	3	5	2	2	2	2	3	2	4
12	80	95.6	4	5	1	0	0	2	2	0	3
13	50	105.6	3	5	2	1	1	2	2	1	3
14	100	90.5	3	5	2	1	0	2	2	1	4
15	90	105.7	6	6	2	1	1	2	1	2	3
16	100	109.9	2	5	2	1	2	2	2	1	4
17	90	111.5	3	5	2	2	2	2	2	1	4
18	90	92.9	3	5	2	2	2	2	3	1	4
19	100	99.8	2	5	2	1	1	2	2	2	4
20	110	97.4	4	6	2	1	1	3	3	2	3
21	120	112.8	3	4	1	1	1	2	2	1	4
22	80	100.7	6	6	2	2	2	2	2	1	3
23	80	103.3	2	5	2	1	1	2	3	2	3
24	90	89.9	3	4	2	1	2	2	2	1	3
25	90	106.4	5	7	2	1	1	2	2	1	4
Mean ± SD	90.0 ± 16.6	101.2 ± 7.6	3.6 ± 1.4	5.3 ± 0.8	1.9 ± 0.3	1.2 ± 0.6	1.1 ± 0.7	2.0 ± 0.2	2.2 ± 0.5	1.1 ± 0.6	3.4 ± 0.6

EBL, estimated blood loss; VAS, visual analog.

### VAS (leg pain and back pain)

The mean of postoperative VAS of back pain at 1 (2.0 ± 0.2), 3 (2.2 ± 0.5), and 6 (1.1 ± 0.6) months postoperatively were lower compared to the preoperative. Median leg pain VAS scores were 1 (1.9 ± 0.3), 3 (1.2 ± 0.6), and 6 (1.1 ± 0.7) months postoperatively.

### Screw accuracy

A total of 116 screws were inserted using robotic navigation. There was only one lateral breach (0.8%) and one superior breach (0.8%). No medial or inferior breaches were observed during the follow-up period.

### Operating time

The operating time for single-position OLIF and percutaneous screw insertion from skin incision until skin closure was 101.2 ± 7.16 min (Table 2). The operation was divided into four steps. The first is the application of dynamic reference based on the iliac wing and registering the CT scan data into the Excelcius GPS; this procedure took around 31.3 ± 8.5 min. The second procedure was the insertion of percutaneous pedicle screws; every single screw needed 3.4 ± 1.6 min, and the total time for single-level percutaneous screws was 13.6 ± 8.5 min. The third step was doing the OLIF procedure; it took around 41.5 ± 8.2 min for a single level, and for every other level above or below, it needed an additional 20.2 ± 4.3 min. The final step was inserting the rod into the screw; it took around 8.3 ± 2.3 min for bilateral rod placement.

### Estimated blood loss

The estimated blood loss was 90.0 ± 16.6 ml (range 50–120 ml).

### Post operative length of stay (LOS)

The mean hospital stay for each patient was 3.4 ± 0.6 days; all patients were discharged after being fully mobile without any walking aid and without the need for high doses or strong analgesics.

### Complications

There were two (8%) vascular complications during surgery; the common iliac vein was accidentally torn during mobilization for access to the L4/5 disc. It was managed using absorbable hemostatic application over the ruptured vein. One patient (4%) had retrograde ejaculation after fusion of L5/S1; the symptom resolved within 3 months after the surgery. In this study, the patient who experienced retrograde ejaculation is different with the patient who experienced major venous bleeding. Of the 116 screws that were inserted using robotic navigation, there were one lateral breach (0.8%) and one superior breach (0.8%). No medial or inferior breaches were observed during the follow-up period.

### Illustrative case

#### Case 1

**Figure 7:**
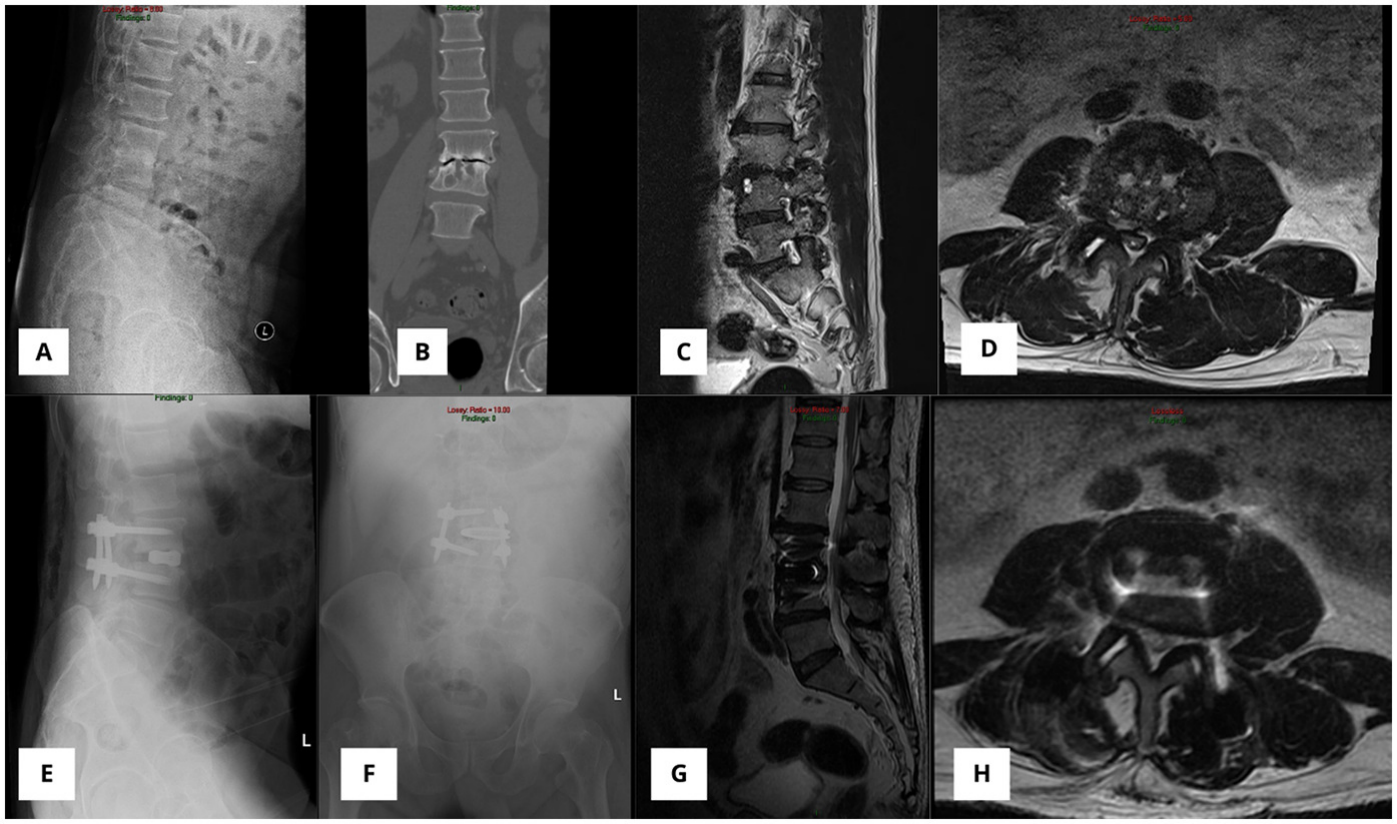
**(A)** X-ray image in lateral extension showing disc narrowing in L3/4 before surgery. **(B)** CT scan showing disc narrowing in L3/L4. **(C,D)** MRI showed disc narrowing at the level of L3/4. Postoperative x-ray **(E,F)** and MRI **(G,H)** showed that the intervertebral cage was well positioned without displacement.

A 53-year-old male underwent an L2–4 OLIF for chronic low back pain due to degenerative disc disease (Figures 7A–D). Preoperatively, he reported a VAS score of 2 for leg pain and 5 for back pain. A robotic-assisted OLIF procedure was performed with a lateral decubitus approach. No intra and postoperative complications were found in this patient. The patient's symptoms resolved until 6 months after surgery (Figures 7E–H).

#### Case 2

**Figure 8:**
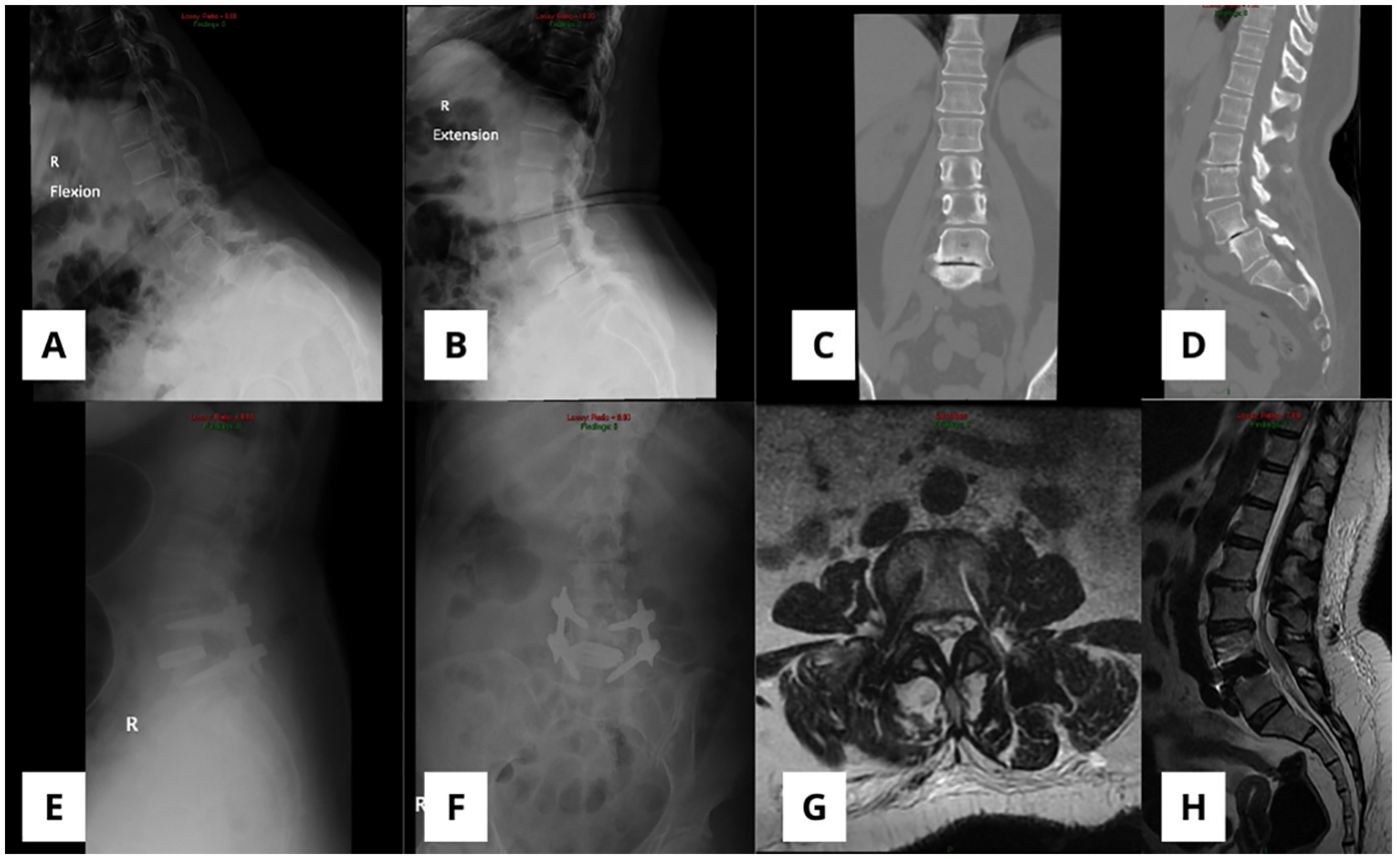
**(A,B)** X-ray image in lateral flexion and extension showing spondylolisthesis L4/5 before surgery. **(C,D)** CT scan showed disc narrowing at the level of L5/S1. Postoperative x-ray **(E,F)** and MRI **(G,H)** showed that the intervertebral cage was well positioned without displacement.

A 70-year-old female underwent a one-level L4/5 robotic-assisted OLIF surgery. She had been experiencing leg pain and lower back pain for the past 3.5 years, with moderate to severe preoperative leg pain (VAS 6) and back pain (VAS 7). Imaging studies, including plain radiographs and a CT scan, revealed spondylolisthesis at L4/5 (Figures 8A–D). After thorough consideration, a robotic-assisted OLIF procedure was performed using a lateral decubitus approach. The patient's hospital stay was 3 days. Postoperative imaging confirmed a successful surgery with no evidence of any breaches (Figures 8E–H). At a follow-up visit 6.2 months later, her leg and back pain had significantly improved, with VAS scores of 1.2 and 0, respectively. No complications were reported intraoperatively or postoperatively.

## Discussion

Robotic surgery has become widely accepted as a treatment modality in medicine. Spine surgery is also part of the medical field that has already accepted the technology (6). All surgeries included in this retrospective study were performed by a single surgeon, using a consistent technique throughout. A small margin of error for pedicle screw insertion that could lead to devastated neurologic or vascular injury is the reason why this technology is becoming more and more popular in the field of spine surgery (7). Robotic navigation offers two key benefits in lateral lumbar surgery. First, it allows the surgeon to easily insert percutaneous pedicle screws in the lateral position without the need for a C-arm. Second, it can be used with robotic instruments for endplate preparation and interbody cage implantation. A robotic arm can also function as a retractor holder, providing more excellent stability than a table-mounted retractor.

Navigation spine surgery with a robotic arm could increase time efficiency during operation (8). Traditionally, the interbody cage was inserted in a lateral decubitus position for OLIF or XLIF procedure, and the patient was flipped to prone for insertion of a pedicle screw (9). The robotic arm provides a rigid platform that enables to perform pedicle screw insertion in the lateral decubitus position. In this study, we did not compare the operating time between a single position and lateral then prone (bipedal position). However, time efficiency had already been shown by another study by Ziino et al., which showed a statistically significant time difference between the two procedures (10). A study comparing robotic-assisted OLIF in single vs. bipedal positions found that the single position resulted in a shorter operative time, higher screw accuracy at the A level, and lower back pain VAS scores at one week postoperatively compared to the bipedal position (11). Tong et al. found a significantly different VAS between robotic and freehand in three days postoperative. However, no significant difference was found in the three- and six-month follow-ups (12).

Our data found that no medial pedicle breaches were using robotic spine navigation. Laine et al. categorized pedicle screw breaches into four types: medial, lateral, superior, and inferior, based on the location of the breach (13). Due to the anatomical proximity between the pedicle and the corresponding nerve root, medial or inferior breaches carry a high risk of nerve injury (14). In contrast, lateral or superior breaches pose a lower risk (15).

Another data comparing free hand and navigated screw insertion showed that the possibility of misplacement in the free hand group is relatively high, ranging from 3% to 55% in the thoracic spine and 5%–41% in the lumbar spine (16–18). Pedicle wall breaches in the lateral and superior parts could be caused by soft tissue pressure in the robotic instrument or skiving of the pilot drill on the lateral wall of the facet joint (19). The haptic feedback that is received from the instrument, combined with the trajectory in the navigation monitor, is essential in maintaining accuracy in pedicle screw insertion (20).

Intraoperative blood loss is reduced in robotic-assisted OLIF surgery compared to the freehand technique due to the smaller incisions required in robotic-assisted procedures. The freehand technique typically involves longer operative times, contributing to increased estimated blood loss (21). Similarly, Several studies have demonstrated that OLIF enables aggressive correction of spinal deformities, reduces postoperative back pain, achieves higher fusion rates with thorough disc space clearance, and decreases blood loss (22–24).

The most commonly reported complications of OLIF surgery in the literature include vascular, ureteral, and nerve damage, as well as lower extremity weakness (25). Other complications reported include male sexual dysfunction, cerebrovascular accidents, peritoneal laceration, ileus, psoas paresis, and groin numbness (22). In our cases, there were two vascular complications during surgery and one instance of retrograde ejaculation post-surgery. While significant vascular injuries are rare in OLIF, careful preoperative planning that takes vascular anatomy into account is essential to prevent such catastrophic events (26). Bifurcation of the abdominal aorta occurs commonly on the L4 level (67%–83%), but various anatomies can be found at the L3 vertebral body level (27, 28). As a result, the iliac artery is already present at level L4 and lies near the psoas major muscle. During procedures, mobilization is made to change the patient's position, which can cause venous bleeding. The surgeon manages the bleeding using patch methods, and the bleeding stops (29).

Retrograde ejaculation, believed to result from injury to the superior hypogastric nerve plexus, is rarely reported. To minimize the risk of postoperative sexual dysfunction, especially in male patients, meticulous blunt dissection and gentle retraction of structures within the great vessel bifurcation are essential (30). Tannoury et al. reported on 940 patients and 2,429 fusion levels using the OLIF technique. The overall surgical complication rate was 8.2%, with no instances of major vascular injury (31). Xiao-guang, et al. reported complications associated with robotic-assisted OLIF procedures. These complications included sympathetic nerve injury and transient thigh flexion weakness or numbness, all of which resolved within the first three months. These issues arose due to the anatomical positioning of the lumbar plexus, lumbar sympathetic trunk, and segmental arteries located laterally in front of the lumbar vertebrae, making them vulnerable to irritation or injury (32).

Although single-position surgery is not a new concept and is widely practiced in lateral or prone positions across many hospitals, integrating robotic assistance in oblique lateral interbody fusion (OLIF) significantly enhances surgical precision, safety, and efficiency. Robotic guidance allows accurate preoperative planning and real-time navigation, which is especially important given the complex retroperitoneal anatomy near the psoas, major vessels, and lumbar plexus. It improves implant accuracy, minimizes risk to critical structures, reduces radiation exposure, and supports minimally invasive techniques for better outcomes.

This study has several limitations. First, the absence of a control group restricts the ability to draw comparative conclusions or establish causality. The primary objective was to assess trends and feasibility within a specific patient population, making a control group beyond the study's scope. Second, validated outcome measures like the Oswestry Disability Index (ODI) were not used due to inconsistent data availability, limiting standardized assessment of functional outcomes. Additionally, the relatively short follow-up period may not capture long-term complications. The surgeries were also performed by four different spine surgeons, introducing potential variability in technique and outcomes. Future studies should include longer follow-up, larger sample sizes, and standardized measures to enhance result reliability. Lastly, this study did not include statistical analysis. The primary objective was to describe the surgical technique and evaluate the feasibility and safety of robotic-assisted oblique lumbar interbody fusion (OLIF), rather than to perform hypothesis-driven comparisons.

## Conclusions

Single-position OLIF shows favorable results, with robotic guidance offering substantial benefits, including reduced bleeding, fewer surgical complications, lower VAS, and shorter operative times without repositioning the patient.

## Data Availability

The original contributions presented in the study are included in the article/Supplementary Material, further inquiries can be directed to the corresponding author.
